# Supplementary material for: Experience of Isavuconazole as a Salvage Therapy in Chronic Pulmonary Fungal Disease
Source: J Fungi (Basel). 2022 Mar 31;8(4):362. doi: 10.3390/jof8040362 (PMC9029347; doi:10.3390/jof8040362)
Supplement: Supplementary file 1 [file jof-08-00362-s001.zip › figure S1.pdf]

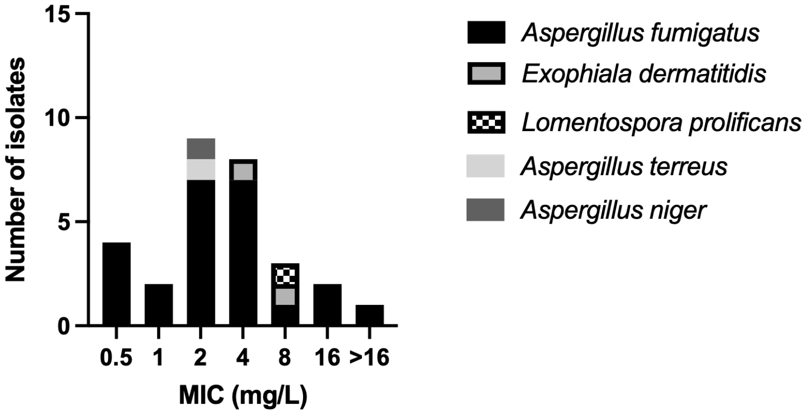

Figure S1. Distribution of isavuconazole MIC (*x* axis, in milligrams per litre) against *Aspergillus* spp., *Lomentosprora prolificans,* and *Exophiala dermatitidis* clinical isolates throughout study period (total 29 clinical isolates).
